# Molecular Mechanism and Pathogenesis of Sarcopenia: An Overview

**DOI:** 10.3390/ijms22063032

**Published:** 2021-03-16

**Authors:** Anna Picca, Riccardo Calvani

**Affiliations:** 1 Fondazione Policlinico Universitario Agostino Gemelli IRCCS, 00168 Rome, Italy; riccardo.calvani@guest.policlinicogemelli.it; 2 Aging Research Center, Department of Neurobiology, Care Sciences and Society, Karolinska Institutet and Stockholm University, 17165 Stockholm, Sweden

Sarcopenia involves a progressive age-related decline of skeletal muscle mass and strength/function [1]. This muscle loss is associated with increased risk of adverse health outcomes, including falls, morbidity, loss of independence, disability, and mortality [2]. Among the processes contributing to age-related muscle wasting, altered hormonal status, chronic inflammation, redox imbalance, loss of α-motor neurons, muscular mitochondrial dysfunction, altered myocyte autophagy, accelerated apoptosis of myonuclei, and impaired satellite cell function are believed to be major factors [3]. Of note, some of these factors belong to the so-called hallmarks of aging, which allows sarcopenia to be framed as a geroscience condition [4]. 

The Special Issue “Molecular Mechanism and Pathogenesis of Sarcopenia” was conceived as an editorial project to bring together basic researchers and clinicians working in the area of sarcopenia in humans and animal models to gain insights into the molecular mechanisms involved in its pathophysiology and provide an appraisal of the state of the art on sarcopenia biomarkers. 

Factors spanning muscle-specific processes (e.g., mitochondrial dysfunction in skeletal myocytes) and systemic mediators pertaining to various domains (e.g., inflammation and amino acid dysmetabolism) have been pinpointed as candidate molecular markers of sarcopenia [5]. The search for specific mediators that may allow early identification and tracking of the condition is very active and continuously evolving [6,7,8]. 

Mitochondrial dysfunction has been identified as a core mechanism in skeletal muscle aging and sarcopenia [9]. This tight relationship seems to be mainly built upon the strong reliance of skeletal muscle cells on oxidative metabolism that makes them highly susceptible to the detrimental effects of overproduction of reactive oxygen species (ROS) as a bioproduct of their metabolism [10]. This is especially relevant in sarcopenic muscles in which dysmorphic, ROS-producing mitochondria are inefficiently cleared and accumulate within cells [11]. These features have also been identified in cells acquiring a senescent phenotype [12]. Indeed, the presence of great amounts of mitochondrial ROS in skeletal muscle cells with accumulation of single-strand breaks in telomere regions may accelerate telomere erosion and trigger cellular senescence [13]. Moreover, counteracting mitochondrial ROS generation has been shown to decrease the rate of telomere shortening, extend muscular cells survival, and restore muscle homeostasis [14,15], ultimately delaying the onset of sarcopenia. For these reasons, the maintenance of a redox balance is a potential intervention for counteracting sarcopenia [16]. Exercise training under different regimens is among the few effective strategies for preserving muscle health in old age by regulating several processes at molecular, cellular, and organismal levels [16,17]. Besides scavenging oxidant species, in middle- to old-aged mice, exercise extends lifespan and healthspan through changes in the muscle transcriptome and metabolism also via the modulation of the pro-longevity Cisd2 gene [17].

The adaptive mechanisms that become compromised during muscle aging are also shared with other pathological conditions, including non-alcoholic fatty liver disease (NAFLD), heart failure (HF), and autoimmune and rheumatic diseases [18], for which the identification of the mechanisms underlying sarcopenia would be valuable [18,19,20,21,22]. The accrual of ROS-induced damage to biomolecules observed in NAFLD has been associated with a progressive loss of liver function [18]. In this context, altered integrated stress response system involving mitochondrial unfolded protein response (UPR^mt^) and mitophagy have been involved in disease development [18]. Indeed, while prolonged exposure to low levels of stress may trigger these processes to maintain mitohormesis, their uncontrolled chronic activation may lead to cell death [18]. 

On a similar note, chronic low-grade inflammation and the associated changes in body composition and declining physical function [23,24,25] are shared features between sarcopenia and autoimmune and rheumatic diseases [21]. In particular, the installment of a pro-inflammatory milieu mainly involving interleukin (IL) 1β, IL6, and tumor necrosis factor-α (TNF-α) has been identified in rheumatoid arthritis (RA) and indicated as a pathogenic mechanism in the disease [21]. Notably, these cytokines are also characteristic of RA patients with sarcopenia and high resting energy expenditure [26,27]. The existence of such a relationship has led to hypothesize that the inflammatory response mounted in RA may favor sarcopenia. Results from preclinical models have shown that muscle wasting during RA occurs as a consequence of the disease itself, rather than as a result of decreased mobility [28]. However, the mechanisms underlying muscle wasting in RA are unclear. A possible explanation may reside in an excessive activation of proteolysis driven by a catabolic response instead of decreased myogenesis [29]. Indeed, rats with adjuvant-induced arthritis, a model of arthritis-induced muscle wasting, showed higher expression of IL1β, E3 ubiquitin ligases atrogin-1 and muscle RING-finger 1 (MuRF1), phosphorylated p38 mitogen-activated protein kinase (MAPK)/p38 MAPK, and nuclear factor kappa-light-chain-enhancer of activated B cells (NF-κB) [27]. NF-κB and p38 MAPK are known activators of the ubiquitin proteasome system [30] and this pro-atrophy signaling pathway in RA may be triggered by IL1β signaling [27]. However, the expression of the myogenic regulators MyoD, paired box 7, and myogenin was also increased in RA rodents with muscle wasting [27]. Therefore, concomitant muscle repair or anabolic compensation may be triggered in arthritis-induced muscle wasting [27]. Along similar routes, skeletal muscle wasting develops in patients with chronic HF in whom it represents a strong predictor of frailty and reduced survival [20].

Chronic inflammation has also been indicated as a molecular link between gut dysbiosis and muscle wasting in old age [31,32]. Albeit the determinants of this crosstalk are not fully elucidated, the adoption of multi-marker approaches combined with multivariate modeling allowed identifying patterns of circulating inflammatory, metabolic, and microbial mediators that may accurately identify older adults with physical frailty and sarcopenia (PF&S) [7,33]. These consist of higher serum levels of aspartic acid, lower circulating concentrations of threonine, and the macrophage inflammatory protein 1α. The levels of these serum mediators were associated with increased abundance of *Oscillospira* and *Ruminococcus* microbial taxa, and decreased abundance of *Barnesiellaceae* and *Christensenellaceae* in older people with PF&S [33]. Further to this, Ticinesi et al. [34], in a proof-of-concept study, explored the existence of a gut-muscle axis in older adults with low muscle mass and low performance by exploring fecal microbiota composition and function with shotgun metagenomics sequencing [34]. Older adults with sarcopenia showed significant depletion of *Faecalibacterium prausnitzii*, *Roseburia inulinivorans* and *Alistipes shahii* microbial species that are known for their metabolic capacity of producing short-chain fatty acids (SCFAs) [34]. The analysis of the fecal metagenome also showed a different representation of genes belonging to several metabolic pathways, including depletion of genes involved in SCFA synthesis, biotransformation of carotenoid and isoflavone, and amino acid interconversion [34].

Albeit a fairly large number of metabolic, microbial, and inflammatory biomolecules have been investigated for their association with PF&S, a “gold standard” biomarker that can reliably predict functional impairment in older adults is still missing. Clinical, functional, and imaging parameters are still reference standards for the identification of PF&S while the incorporation of biological markers into clinical practice has yet to be achieved. Multi-platform regression methods developed to handle highly correlated variables have shown great potential for addressing the complexity of PF&S pathophysiology and unveiling novel targets for interventions. Well-designed longitudinal studies are highly sought after to accomplish these ambitious tasks.
